# Predictive factors for difficult mask ventilation in the obese surgical population

**DOI:** 10.12688/f1000research.5471.1

**Published:** 2014-10-09

**Authors:** Davide Cattano, Anastasia Katsiampoura, Ruggero M. Corso, Peter V. Killoran, Chunyan Cai, Carin A. Hagberg

**Affiliations:** 1 Department of Anesthesiology, University of Texas Medical School at Houston, Houston, TX 77030, USA; 2 Department of GI Medical Oncology, MD Anderson Cancer Center Hospital, Houston, TX 77030, USA; 3 Emergency Department, Anesthesia and Intensive Care Section, GB Morgagni-L.Pierantoni Hospital, Forli, 47121, Italy; 4 Division of Clinical and Translational Sciences, Department of Internal Medicine, The University of Texas Medical School at Houston, Houston, TX 77030, USA

## Abstract

**Background**

Difficult Mask Ventilation (DMV), is a situation in which it is impossible for an unassisted anesthesiologist to maintain oxygen saturation >90% using 100% oxygen and positive pressure ventilation to prevent or reverse signs of inadequate ventilation during mask ventilation.  The incidence varies from 0.08 – 15%. Patient-related anatomical features are by far the most significant cause.  We analyzed data from an obese surgical population (BMI> 30 kg/m ^2^) to identify specific risk and predictive factors for DMV.

**Methods**

Five hundred and fifty seven obese patients were identified from a database of 1399 cases associated with preoperative airway examinations where mask ventilation was attempted. Assessment of mask ventilation in this group was stratified by a severity score (0-3), and a step-wise selection method was used to identify independent predictors.  The area under the curve of the receiver-operating-characteristic was then used to evaluate the model’s predictive value. Adjusted odds ratios and their 95% confidence intervals were also calculated.

**Results**

DMV was observed in 80/557 (14%) patients. Three independent predictive factors for DMV in obese patients were identified: age 49 years, short neck, and neck circumference  43 cm. In the current study th sensitivity for one factor is 0.90 with a specificity 0.35. However, the specificity increased to 0.80 with inclusion of more than one factor.

**Conclusion**

According to the current investigation, the three predictive factors are strongly associated with DMV in obese patients. Each independent risk factor alone provides a good screening for DMV and two factors substantially improve specificity. Based on our analysis, we speculate that the absence of at least 2 of the factors we identified might have a significant negative predictive value and can reasonably exclude DMV, with a negative likelihood ratio 0.81.

## Introduction

Bag mask ventilation commonly precedes the establishment of a secure airway by endotracheal intubation. However, the degree of difficulty encountered is variable ^1– 4^, with the incidence of Difficult Mask Ventilation (DMV) varying from 0.08–15% depending on the criteria used for the definition. The American Society of Anesthesiologists’ (ASA) original definition recognized DMV as a situation where it is not possible for the unassisted anesthesiologist to maintain the oxygen saturation > 90% using 100% oxygen and positive pressure ventilation, or to prevent or reverse signs of inadequate ventilation ^5^. Subsequently, many other definitions have evolved taking into account patient-independent factors that contribute to DMV, such as provider--and equipment-related factors ^5^. Moreover, as an effort to overcome subjective definitions, several grading scales have been proposed, including Adnet’s and Han’s scales ^1, 6^.

In the face of DMV, critical hypoxemia may rapidly ensue and emphasizes the need for proper identification of risk factors during the preoperative assessment. Obese patients remain one of the most challenging patient populations for airway management ^7– 9^, with difficulties arising due to both anatomical features and functional changes ^10, 11^. Current protocols for preoperative evaluation focus not only on anatomic characteristics, but also on the identification of systemic features that are associated with airway obstruction and physiologic disarrangements, such as obstructive sleep apnea syndrome (OSA) ^8, 12^. For instance, in the general surgical population, a history of OSA has been found to be an independent risk factor of impossible mask ventilation ^13, 14^, and patients with a high BMI have a high risk for OSA ^12^. However, despite the known association between DMV, obesity, and OSA, there are no established predictive criteria, nor a simple scoring system which could predict DMV in the obese population.

In the present investigation, we primarily aimed to identify specific risk and predictive factors for difficult mask ventilation in obese patients and secondarily we attempted to correlate history and predicted factors related to OSA with DMV. We performed a retrospective analysis based on an existing database ^14^.

## Methods

A retrospective investigation was performed to identify predictive markers of DMV in obese patients at Memorial Hermann Hospital-Texas Medical Center utilizing an existing database of airway assessment and airway management records ^4, 14^ : 1399 anesthetics were identified where both mask ventilation was attempted and a pre procedure airway evaluation was documented. Of these, 557 obese patients were identified and included for analysis. The preoperative assessment utilized a dedicated airway assessment form ^14^ which included Mallampati pharyngeal classification (modified by Samsoon and Young) ^15^, inter-incisor gap and thyromental distance (cm) measured with the neck extended, sternomental distance, BMI, neck circumference (cm) measured at the level of the thyroid cartilage, dentition status, presence of facial hair, facial or neck trauma, nasal deficiencies, neck mobility grade (which was divided into three categories according to the mouth-occiput distance), diagnosis of OSA according to patient history, perceived short neck, history of difficult intubation, and cervical spine abduction. Due to the retrospective nature of the study we were able to assess the OSA status only by the patient history. The degree of DMV classified by the provider performing the case by a severity score ^1^: 0 = easy, 1 = oral airway used, 2 = two handed ventilation and 3 = extraglottic device required. Based on the severity, mask ventilation was considered True DMV if the ease of mask ventilation was graded as 2 or 3 and False DMV if it was graded as 0 or 1. During attempts at mask ventilation, all obese patients were placed in the head elevated laryngoscopy position and the operating room table was titled in the reverse Trendelenburg position. Vital signs were monitored according to ASA standard general anesthesia monitoring. Neuromuscular blocking agent utilization and/or the time of administration, dosage and reason for administration was not captured in the source database and therefore not included in this retrospective investigation.

## Statistical analysis

Statistical analyses was performed using SAS 9.3 (SAS Institute, Cary, NC, USA). A p-value <0.05 was considered significant. Obese patients with or without DMV were compared. Values were reported as mean ± standard deviation for continuous variables and frequency (percentage) for categorical variables for all preoperative patient characteristics. First, a univariate comparison between patients with or without DMV was performed using two sample t-test for continuous variables and Chi-square test or Fisher exact test, as appropriate, for categorical variables. Age was dichotemized based on a threshold of 49 years and neck circumference of 43 (cm), based on recognized risk threshold ^7^. All variables with a p-value <0.20 in univariate analysis were entered into a multivariate logistic regression model. Stepwise selection method was used to identify independent predictors of DMV. All variables that were statistically significant with a p < 0.05 were established as independent predictors. Age and neck circumference were dichotomized according to clinical suggestions, using the optimal cut-off value identified by maximizing the sum of sensitivity and specificity for the primary outcome to obtain the best accuracy. In addition, the area under the curve of the receiver-operating-characteristic was calculated to evaluate the resulting model’s predictive value. The adjusted odds ratios and their 95% confidence intervals were also calculated.

## Results

A total of 557 cases of attempted mask ventilation were recorded in obese patients, as shown in Table 1, of which 78 were considered to be DMV (14.3%). Patient characteristics and statistical correlations between DMV and preoperative variables are presented in Table 2.

**Table 1.** Summary statistics for MVEase.

MVEase	Frequency (percentage) N=557
0	267 (47.9)
1	210 (37.7)
2	77 (13.8)
3	3 (0.5)

Define DMV=True if MVEase=2,3 and DMV=False if MVEase=0,1.

**Table 2.** Preoperative patient characteristics by DMV status.

Variables	DMV		p-value
	False (MVEase=0,1) N=477	True (MVEase=2,3) N=80	
**Age (year)** ≥49	45.6±15.0 197 (41.3)	48.4±13.2 47 (58.8)	0.124 0.004
**Male**	206 (43.2)	44 (55.0)	0.049
**BMI (kg/m ^2^)**	36.5±5.6	37.7±6.1	0.091
**NeckCirc** ≥43	41.8±4.6 213 (44.7)	44.4±4.5 53 (66.3)	<0.0001 0.0003
**InterIncisors**	4.8±0.9	4.8±0.9	0.768
**Thyromental**	7.9±1.7	8.0±1.7	0.724
**Sternomental**	15.3±2.3	15.2±2.0	0.738
**HxDiffIntub**	2 (0.4)	0 (0)	NR
**NeckMobGrade** 1 2,3	424 (88.9) 53 (11.1)	65 (81.3) 15 (18.8)	0.053
**Mallampati** I, II III, IV	382 (80.1) 95 (19.9)	62 (77.5) 18 (22.5)	0.595
**CSpineAbn**	14 (2.9)	5 (6.3)	0.172
**NoTeeth**	29 (6.1)	10 (12.5)	0.037
**FacHair**	43 (9.0)	12 (15.0)	0.097
**FacTrauma**	4 (0.8)	0 (0)	NR
**FullStomach**	3 (0.6)	1 (1.3)	0.543
**NasalDef**	1 (0.2)	1 (1.3)	0.267
**NeckTrauma**	3 (0.6)	2 (2.5)	0.153
**ShortNeck**	53 (11.1)	21 (26.3)	0.0002
**ObsSA**	108 (22.6)	29 (36.3)	0.009
**ResYear** CA-1, CA-1-2 CA-2, CA-2-3, CA-3	362 (75.9) 115 (24.1)	60 (75.0) 20 (25.0)	0.863

NR: not reported due to zero cells. Values are reported as mean±SD and frequency (percentage).

Based on a univariate analysis, a total of 6 factors were identified with a p value < 0.05 including: age, gender, neck circumference, absence of teeth, short neck (subjective) and ΟSA (suspected or diagnosed). Thresholds used were based on clinical suggestions. Age was dichotomized based on a threshold of 49 years of, and neck circumference based on, 43 cm. Incorporation of these 6 factors into a multivariate logistic regression model identified 3 independent predictive factors for DMV in obese patients. The model used step-wise selection and identified age ≥ 49 years, short neck, and neck circumference ≥ 43 cm ( Table 3) as statistically significant. OSA, gender, and absence of teeth were not considered significant in the multivariate model.

**Table 3.** Independent predictors of difficult mask ventilation by multivariate logistic regression model.

Predictor	β Coefficient	Standard Error	*P* value	Adjusted odds ratio (95% Confidence Interval)
Age ≥ 49	0.707	0.251	0.005	2.03 (1.24, 3.32)
NeckCirc ≥ 43	0.804	0.259	0.002	2.23 (1.35, 3.71)
Short Neck	0.975	0.302	0.034	2.65 (1.47, 4.79)

Although a total of 3 risk factors were identified, no individual subject had more than 2 risk factors. The 3 independent risk factors identified were then applied to all cases where DMV was encountered to evaluate a predictive model for DMV in obese patients. The sensitivity, specificity, likelihood ratios, and predictive values were progressively calculated for patients with different numbers of risk factors. The adjusted odds ratios were analyzed ( Table 4).

**Table 4.** Diagnostic value of the cut-off for number of risk factors in predicting a difficult mask ventilation.

Cut-off for number of risk factors	Sensitivity	Specificity	Likelihood ratio positive	Likelihood ratio negative	Positive predictive value	Negative predictive value
1	0.90	0.34	1.36	0.29	0.19	0.95
2	0.35	0.80	1.75	0.81	0.23	0.88

Likelihood ratio positive=Sensitivity/(1-Specificity) Likelihood ratio negative=(1-Sensitivity)/Specificity.

Table 4 displays the sensitivity and specificity if we use the given value of the number of risk factors possessed by patients as a cut-off to classify DMV. For example, when we use number of risk factors at 1 as a cut-off, i.e., any patients with >=1 risk factors will be classified as DMV=1 and any patients with <1 risk factors will be classified as DMV=0, the sensitivity will be 0.90 and specificity will be 0.34. Cut-off at 1,2 are calculated and displayed.

**Figure 1.**
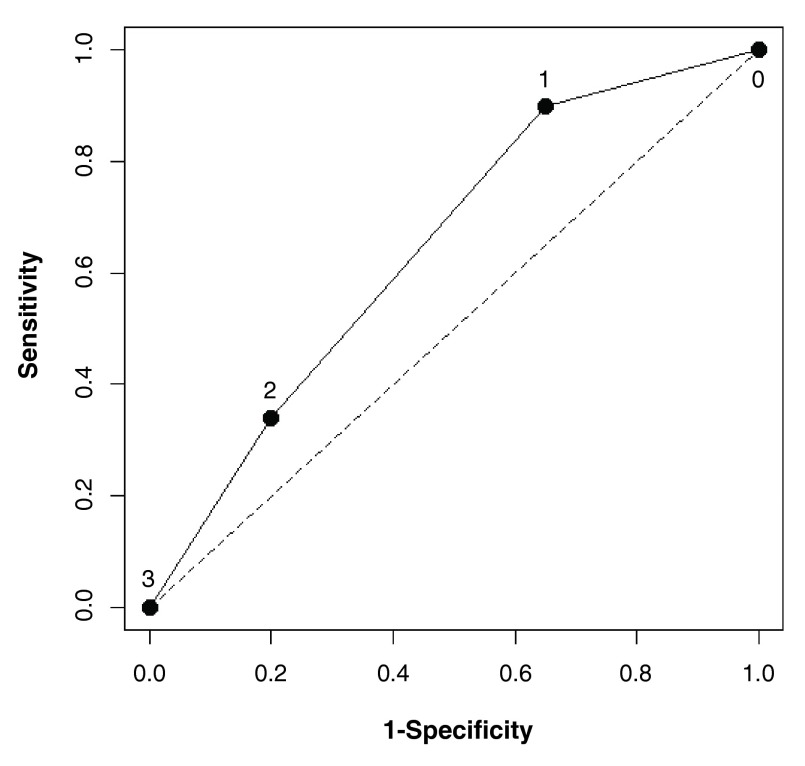
A receiver-operating-characteristic (ROC) curve evaluating the sensitivity and specificity of preoperative independent risk factors for difficult mask ventilation for BMI>=30 patients. Three independent predictors for difficult mask ventilation were identified using logistic regression: age of 49 yr or older, NeckCirc of 43 or greater, and Short Neck. The area under the curve was 0.65 (95% confidence interval: 0.59 – 0.70).

A ROC curve ( Figure 1) evaluating the sensitivity and specificity of preoperative independent risk factors for DMV for BMI>30 kg/m ^2^ patients was calculated. The model’s c-statistic score was 0.65 with 95% CI of 0.59 to 0.70. The sensitivity for one factor is 0.90 with a specificity of 0.35. However with more than one factor, the specificity increased to the level of 0.80.

## Discussion

We performed a retrospective analysis based on a database of airway assessment and airway management records collected at Memorial Hermann Hospital-Texas Medical Center, a tertiary care center. Our study focused in stratification and the identification of DMV predictive factors in a surgical population of obese patients, while recently we reported DMV in the general population ^4^.

In our cohort, the incidence of DMV in obese patients was 14%. These findings are consistent with previous reports by Leoni and Kheterpal ^7, 13^. In their study, Leoni *et al.* reported that the incidence of DMV is significantly higher in obese patients compared to the general surgical population ^7^. We also compared the incidence of DMV in the obese population to the general surgical population, confirming a frequency of 14% and 8.8 ^4, 9^ respectively. The finding emphasizes the different risk stratification of DMV in the obese patients. Interestingly, in our obese surgical population OSA was as frequent as 24%, while in other studies the prevalence of OSA among bariatric surgery patients reaches up to 70% and, in the general population, is approximately 20% ^16– 18^.

In the present investigation, the statistical analysis identified 3 novel independent predictive markers for DMV in obese population: (a) age ≥ 49 years, (b) neck circumference ≥ 43 cm, and (c) perceived short neck. In the general population 7 risk factors were previously identified, of which OSA and BMI were two of them ^4^: interestingly the latter together with facial hair and history of difficult intubation were not present in the current model. This could be attributed to the reduction of the sample size, to the specific characteristic of obese patient (which are not necessarily at increased risk of difficult intubation) ^19^ or the effect of the stratification used which could mask the effect of BMI and OSA.

Based on our analysis, we speculate that the absence of at least 2 of the factors we identified might have a significant negative predictive value and can reasonably exclude DMV, with a negative likelihood ratio 0.81. To our knowledge, this is the first time that short neck and age ≥ 49 years are recognized as risk factors for DMV specifically in obese patients; however this is not totally unexpected. According to Langeron *et al.*, age >55 years is correlated with DMV in the general population ^20^, thus it seems reasonable that age would be a risk factor for DMV in the obese population as well. Shah *et al.* consider short and thick neck as an independent risk factor in the general population ^21^. Neck circumference could be correlated to anatomical and physiological changes due to obesity that may increase the airway obstruction. Indeed the increased neck circumference is reflecting the presence of excessive palatal and pharyngeal soft tissue which intensifies the collapse of oropharynx during muscle relaxation. As a result increased neck circumference can make mask ventilation more difficult ^22, 23^.

Numerous prospective and retrospective clinical studies examined the correlation of patient-dependent and patient-independent characteristics, along with DMV, in the general surgical population ^8, 20, 24^, and led to the identification of several predictive factors for DMV. Specifically, Langeron *et al.* (as previously stated), Yildiz *et al.* and Kheterpal *et al.* demonstrated that increased BMI, history of snoring or OSA, as well as age ≥ 55 years are risk factors for DMV in the general surgical population ^13, 20, 24^. Additional factors in these studies included the presence of beard, Mallampati classification of III or IV, limited mandibular protrusion test, male gender, and airway masses or tumors. In our investigation, a total of 6 predictive markers of DMV were identified. However, Mallampati classification, limited mandibular protrusion and male sex did not reach significant correlation to DMV (step-wise analysis). All these findings are summarized in Table 5.

**Table 5.** Independent predictors for DMV in general surgical population and obese patients.

Langeroon *et al.* (General population) ^20^	Khetepal *et al.* (General population) ^8^	Cattano *et al.* (General population) ^9^	Leoni *et al.* (Obese) ^7^	Our model (Obese)
**Age 55 yr** 2.26 (1.34–3.81) 0.002	**Male sex** 3. 3 (1.8–6.3) 0.001	**Age ≥ 47** 1.97 (1.32–2.94) 0.001	**Male gender** 1.55 (0.97–2.46) 0.061	**Age ≥ 49** 2.03 (1.24–3.32) 0.005
	**Neck radiation changes** 7.1 (2.1–24.4) 0.002	**Neck Circ ≥ 40 cm** 2.54 (1.59–4.05) <0.001	**Neck circumference** 1.17 (1.08–1.27) <0.0001	**Neck Circ ≥ 43 cm** 2.23 (1.35–3.71) 0.002
**Lack of teeth** 2.28 (1.26–4.10) 0.006	**Mallampati III or IV** 2.0 (1.1–3.4) 0.014	**Hx Difficult intubation** 4.65 (1.20–18.02) 0.026	**Mallampati classification** 2.54 (1.18–3.85) 0.009
**History of snoring** 1.84 (1.09–3.10) 0.02	**Sleep apnea** 2.4 ****(1.3–4.3) 0.005	**OSA** 1.65 (1.07–2.56) 0.023	**Limited jaw protrusion** 1.98 (1.03–4.28) 0.046
**Presence of beard** 3.18 (1.39–7.27) 0.006	**Presence of beard** 1.9 (1.1–3.3) 0.024	**Facial Hair** 2.34 (1.43–3.83) <0.001		
**Body mass index (BMI)** **26 kg/m ^2^** 2.75 (1.64–4.62) 0.001		**BMI ≥ 35 kg/m ^2^** 2.09 (1.35–3.23) 0.001		
		**Short Neck** 1.88 (1.06–3.32) 0.023		**Short Neck** 2.65 (1.47–4.79) 0.034

Adjusted odds ratios with 95% Confidence intervals and P values are noted respectively.

Last and with our surprise, OSA was not an independent risk factor for DMV in our cohort: this could be explained by the overlap of OSA predictive value with other factors, such as neck circumference, which has been shown to correlate with OSA ^25^.

Few comments need to be reserved for the limitations of the present investigation. First, resident physicians were mostly involved in the study and we assumed that all anesthesiology residents had similar educational skills, based on our recent study ^9^. Another limitation is the fact that the report regarding DMV is based on the subjective nature of the DMV definitions. Third, stepwise selection was sample dependent and may artificially enhance the performance of the model. Fourth, the retrospective nature of our data selection could contribute to bias in this study. Lastly, mask ventilation was assumed to be assessed as per current practice after induction and before muscle relaxation, yet the absence of an objective measure in the study about the status of paralysis and the use of muscle relaxant before or after the assessment of the mask ventilation could have partially affected the results.

## Conclusion

In conclusion, in the present study we demonstrated that (a) age ≥ 49 years, (b) neck circumference ≥ 43 cm, and (c) short neck (perceived) are strongly associated with DMV in obese patients. Thus, we suggest that these patient-dependent factors should be included in the pre-operative assessment to better predict DMV in the obese population. Each one used singularly may provide an efficacious screening tool, while the association of 2 of them may be used to improve specificity. Since the prevalence of obese patients in the surgical population is increasing exponentially, further investigation is warranted that may elucidate the association of (1) patient-derived anatomical and functional characteristics, (2) physician-derived characteristics and (3) equipment characteristics with DMV in obese patient.

## Data availability

Data have been obtained from databases at the Memorial Hermann Hospital, Texas Medical Center, Houston, IRB approval HSC-MS-07-0144. The author can support applications to the Institutional Board to make the data accessible upon individual request. Please forward your requests to Davide Cattano.
