# Supplementary material for: Predictive factors for difficult mask ventilation in the obese surgical population
Source: F1000Res. 2014 Oct 9;3:239. [Version 1] doi: 10.12688/f1000research.5471.1 (PMC4244762; doi:10.12688/f1000research.5471.1)
Supplement: Peer review history

# Peer review: Referee response for version 1

**DOI:** 10.5256/f1000research.5841.r6375

**Published:** 2014-11-07

**Authors:** Mirsad Dupanović

**Affiliations:** 1 Department of Anesthesiology, University of Kansas, Kansas City, USA

I would like to compliment Dr. Cattano and his colleagues for undertaking the investigation on this important topic. Their results confirmed most of the risk factors that other investigators have also found may make mask ventilation difficult (studies cited in the reference section of this manuscript). However, Cattano *et al*. have attempted to go a step further and identify as to how many risk factors need to be present for mask ventilation to be difficult. They had a partial success in that venture. The definition of difficult mask ventilation in this study seems very reasonable and I hope it gets wide acceptance among those that manage airway.

I agree with Dr. Doyle's comment that it needs to be re-emphasized that this was a retrospective study and these results should be re-tested in a well designed prospective study.

I have read this submission. I believe that I have an appropriate level of expertise to confirm that it is of an acceptable scientific standard.

# Peer review: Referee response for version 1

**DOI:** 10.5256/f1000research.5841.r6376

**Published:** 2014-11-05

**Authors:** Peter Szmuk

**Affiliations:** 1 Department of Anesthesiology and Pain Management, University of Texas Southwestern, Dallas, USA

Thank you for the opportunity to review this manuscript and my apologies for the long response time.

This manuscript comes from one of the world leading research centers on difficult airway and deals with a very important and possible life saving topic: the difficult mask ventilation (DMV). Based on a retrospective analysis the authors found that three factors (age > 49, neck circumference > 43 and short neck) are strong predictors for DMV in obese patients and thus, should be included in a pre-operative assessment. Despite the retrospective nature of the study, this screening tool should make an important contribution to the predictability of DMV especially in the obese patients. I would encourage the authors to expand their work into a prospective study.

**Minor comments:**

 

- Methods section:
   
  - 1399 anesthetics were identified to have both mask ventilation and airway evaluation. Please specify over what period and/or how many patients.
  - Please define “obese” patients and clarify the term “nasal deficiencies”.
- I am surprised that the dentition status was not one of the factors involved in the DMV. Is that due to the fact that very few patients in your group are edentulous? This might be different in other geographical areas. Could you comment on that?
- Ethnicity and body fat distribution would be another factor to consider in a future study. Obese patients with predominant abdominal fat distribution might have normal neck and airway as compared to those with an equally distributed fat habitus.
- Finally, one of the study limitations noted in the discussion section mentioned the skills level of the residents participating in these cases. This is a valid concern but I would also be interested to see if other factors related to the provider might play a role in DMV. From my clinical observation I noticed that providers with small hands have more difficulty with mask ventilation of a large, obese patient. I wander if anybody looked into this association.

I have read this submission. I believe that I have an appropriate level of expertise to confirm that it is of an acceptable scientific standard.

# Response

**Published:** 2014-11-05

**Authors:** Davide Cattano

**Affiliations:** Department of Anesthesiology, The University of Texas Medical School at Houston, USA

We acknowledge Dr Szmuk comments, and the discussion resulting could further strengthen the data reported. The study was undergone over a period of 18 months, and a selected review of all records with pre- and post intervention data available constituted the cohort analysis. The definition of obesity is the WHO, BMI greater and equal of 30 (kg/m ^2^). We recognize the limitations of a retrospective review and the deficiency in some of the definition, which are clinical indicators though in real world airway management. Indeed in a previous investigation we commented on the inevitable fact that certain predictors remains subjective and dependent on operator assessment. It is interesting that many classification based on standardized definition performs at most with 65% reliability. We agree with Dr Szmuk that body fat distribution is indeed perceived as an important factor. It is interesting that in other studies BMI as low as 26 were found to be associated (general population) and that in European and Asia based study the incidence of DMV would be variable based on other anatomical markers and different body weight, maybe confirming Dr Szmuk's observation.

# Peer review: Referee response for version 1

**DOI:** 10.5256/f1000research.5841.r6608

**Published:** 2014-10-31

**Authors:** Basem Abdelmalak

**Affiliations:** 1 Department of General Anesthesiology, Cleveland Clinic, Cleveland, USA

Thank you for the opportunity to review this very interesting manuscript that focuses on studying difficult mask ventilation in obese patients. This manuscript highlights an important safety consideration when caring for this patient population. The authors have appropriately discussed the limitations of this study. Of those, the most important is the retrospective nature of the study. Another limitation to consider is the lack of data on the use of muscle relaxants: how much and its timing relative to the assessment of the ventilation difficulty, etc. As one might imagine, there may have been variability related to the ongoing controversy of the administration of muscle relaxants either immediately after induction, or a bit delayed till confirmation of the ability to mask ventilate.

As we acknowledge such limitations and thus their impact on the validity of these results, we should keep in mind that the resulting increased awareness of these predictors will likely increase the likelihood of a thorough airway exam and making the right decision in managing such airways. As per the most recent ASA difficult airway algorithm (2013) a thorough airway evaluation will aid in deciding the safest pathway taken inclusive of the following factors:  invasive vs, non-invasive, awake vs. asleep, videolaryngoscopy as first approach vs. DL, and finally maintaining spontaneous ventilation vs. muscle relaxation.

I have read this submission. I believe that I have an appropriate level of expertise to confirm that it is of an acceptable scientific standard, however I have significant reservations, as outlined above.

# Response

**Published:** 2014-10-31

**Authors:** Davide Cattano

**Affiliations:** Department of Anesthesiology, The University of Texas Medical School at Houston, USA

Dr Abdelmalak comments are very appreciated, particularly considering his airway management expertise. The points underlined are significant to strenght discussion about our findings. We would like to comment about the muscle relaxation utilization: as clinical standard at our institution, muscle relaxant administration, the majority of the times, follows bag mask ventilatilability confirmation, but it is also true that several anesthesiologists actually utilize a different pattern (which it has been predicated by expert society as well).

Another important point pertains the predictors. It is interesting to map the risk predictors as per different studies have identified, and knowing the clinical practice pressure we are exposed to, making an effort for the few that seems to have a significant impact may be worth more than being distracted by others.

The last point is related to airway devices and techniques. As standard or routine utilization of certain devices becomes more common, the difficulty of airway management is also evolving. We cannot disregard in this sense the work of Caldiroli and Cortellazzi for instance on the utilization of Glidescope as primary laryngoscopy device: such usage has prompted a revisitation of the El-Ganzouri score, highlighting newer usage of predictive factors because of modification of airway difficulty and outcomes.

Lastly, It is reassuring that several investigations, which ours is one, particularly in different patient populations, are confirming in parallel same findings.
